# Addressing gambling harms by reducing the supply of electronic gambling machines: a comparative study of Italy and Finland

**DOI:** 10.1186/s12889-022-13398-0

**Published:** 2022-05-20

**Authors:** Virve Marionneau, Gabriele Mandolesi, Sara Rolando, Janne Nikkinen

**Affiliations:** 1 Centre for Research on Addiction, Control, and Governance (CEACG), Faculty of Social Sciences, University Researcher, University of Helsinki, Helsinki, Finland; 2 Università Lumsa, Rome, Italy; 3 Research and Training Institute ECLECTICA, Turin, Italy

**Keywords:** Gambling, Italy, Finland, Electronic gambling machines, Total consumption

## Abstract

**Background:**

Electronic gambling machines (EGMs) are amongst the most harmful forms of gambling. The high availability of EGMs is also linked to increased consumption. To reduce the burden of EGMs on public health, policies to reduce their numbers have recently been introduced in Italy and Finland. This study compares the aims and justifications of these legislative changes, as well as their overall impacts on total consumption.

**Methods:**

The objectives and justifications of policies to reduce the number of EGMs were based on qualitative media analysis. The impacts on total consumption were measured using financial figures provided by gambling providers in Italy and Finland.

**Results:**

Results show that the reductions in EGM numbers were justified in terms of public health concerns in both countries, but the amplitude of policies varied. In Italy, the reductions were more ambitious than in Finland, and included reductions in the number of gambling locations. The financial data nevertheless indicated that the reductions may not have been significant enough.

**Conclusions:**

Public health concerns were initially highlighted in the media discussions, but eventually in both countries reduction policies were less ambitious due to industry lobbying and state revenue interests. The reductions therefore do not appear to have been effective in reducing total consumption and the burden on public health.

## Background

Gambling is increasingly understood as a public health issue (e.g., [1, 2]). Harm reduction initiatives should therefore be targeted at the population level. Such initiatives can either aim at promoting less harmful products, or at making harmful products less harmful [3]. The latter approach has recently been taken in Italy and Finland, where the availability of electronic gambling machines (EGMs) has been reduced. Availability reductions, in addition to mandatory identification and limiting product characteristics, are a key policy measure to limit exposure and consumption in gambling [4]. Pricing and taxation (widely used in tobacco and alcohol control, cf. [4, 5]) are less relevant, because particularly EGM and casino gambling have quite low price elasticities of demand [6].

EGMs are amongst the most harmful forms of gambling and EGM gambling is predominant in treatment services for gambling-related problems [3–5, 7–9]. EGMs include a plethora of structural characteristics that reinforce and prolong gambling, such as speed, sensory effects, interactive features, bonuses, near misses, losses disguised as wins (LDWs), and the volatility of pay-outs [4, 7, 10]. The replacement of traditional slot machines with electronic terminals that provide a variety of games is also likely to increase EGM harms [11, 12]. In addition to structural characteristics, situational characteristics, including the availability of EGMs, have been linked to increased consumption and harms. High visibility of EGMs in non-casino environments may further increase their harm potential. A systematic review [9] found that a high availability of EGMs is connected to increased volumes of gambling and increased problem gambling. In Australia, the number of EGM venues in a geographical area has also been connected to personal insolvency cases [13].

The current study compares the policy objectives and justifications of the Italian and Finnish EGM reduction policies and their public health impacts measured in terms of total consumption. Similar to the case of alcohol, gambling harms appear to increase in line with consumption [4, 14]. At the same time, the availability theory [15, 16] suggests that reduced availability reduces total consumption. This theory is also supported by evidence from the EGM closures during COVID-19 [17, 18]. Other perspectives, including gambling restraint erosion theory (GRET) and population adaptation have also been presented, but with less empirical support (see [19, 20], respectively).

The focus on Italy and Finland is justified by the high gambling spending and widespread availability of high-intensity non-casino EGMs in these two contexts. Comparative statistics on EGM numbers [21] in both countries place them amongst those with the highest number of EGMs per capita in Europe. In 2019, mainland Finland had about 21,100 EGMs, of which 18,500 were in non-casino locations and 2600 in gambling arcades. The EGM market in mainland Finland is operated by monopoly holder Veikkaus. (Penningautomatföreningen or PAF, operates on the autonomous Åland islands). In the latest population study, 31% of Finnish past year gamblers had gambled on EGMs within the last 12 months [22]. This is a comparatively high figure.

In Italy, the latest population study from 2017 to 2018 [23] shows that 5.9% of Italian gamblers had gambled on EGMs during the last 12 months. This is notably less than in Finland, but similar to figures from other European countries. Italy had approximately 320,000 EGMs in 2019 (after reductions had already commenced), of which 263,000 were mainly non-casino ‘amusement with prizes’ (AWP) machines, and the remainder were video lottery terminals (VLT) located in arcades. The EGM market in Italy is operated by 11 concessionaries, of which Lottomatica, Sisal, and Snaitech have the largest market shares [24].

Both countries have also recently addressed the harms caused by EGM gambling by initiating reductions in the numbers of EGMs. The Finnish EGM reductions, initiated by the monopoly holder Veikkaus, began in January 2020. The reductions initially set to remove 3500 non-casino EGMs (18% of non-casino machines), but the aim was later set to 8000 (43%). The Italian EGM reduction process was initiated by municipalities concerned over the spread of gambling already in the early 2000s. After discussions at the Permanent Conference on the Relationships between Central Government and the Regions, an agreement was reached in September 2017 to halve the number of gambling venues (from 98,600 to 48,000) and the number of EGMs by 35% (from 400,000 to 265,000) by the end of 2019. However, some pre-existing regional laws are more restrictive [25].

In the following, we introduce material from the literature on the impacts of EGM reductions and analyse the policy change in Italy and Finland. The justifications and objectives of the EGM reduction policies were analysed using qualitative media analysis. The impacts of the reductions on total consumption were measured using financial figures provided by EGM operators.

## EGM reductions in previous research

Impact studies on previous EGM reduction or removal policies have suggested that reduced availability is connected to declining total consumption and problem gambling [4, 9]. Nevertheless, outcomes and impacts have, varied depending on the extent of policies.

First, the magnitude of reductions matters. More important reductions yield better results [4] while a total removal or shutdown of EGMs is connected to the sharpest declines in total consumption. In Norway, the removal of EGMs from public spaces in 2007, and replacement in 2008-2009 with more sparsely situated and less harmful machines reduced gambling consumption and the need for treatment and support services [26–28]. Recent studies conducted on the impacts of COVID-19 on gambling support this observation. The precautions taken to limit the spread of the coronavirus have included EGM shutdowns in many jurisdictions, including Finland and Italy. These shutdowns have lessened the burden of harms caused by EGM gambling, including reduced total consumption and reduced gambling harms [17, 18, 29, 30]. Also, reductions in land-based gambling consumption have not translated into increases in online gambling in many jurisdictions [15, 30, 31].

Second, reducing the number of venues and the number of machines translates into reduced consumption. The frequency of venue visitation and gambling participation have been inversely connected to residential distance from venues [32], suggesting that a sparser network may lead to lower total consumption. In Piedmont, Italy, reductions in EGMs locations and restrictions on opening hours in addition to removing 50% of EGMs was connected to a sharp reduction in gambling consumption, a reduction of at-risk gambling, and a reduction in help-seeking [27, 33].

Third, moving EGMs from non-casino locations such as supermarkets, tobacco shops, and petrol stations to separate gambling arcades may reduce consumption by limiting exposure to gambling. For example, in Poland, the non-casino EGM market was monopolised during 2009-2015. Machines were reintroduced in monopoly-holders’ arcades as of 2018. According to the Polish Ministry of Health [34], limiting EGMs to arcades reduced total consumption. Other research has nevertheless argued that arcades may carry other harm-inducing characteristics. The co-consumption of gambling and alcohol and other venue effects may particularly increase the risk of harms in these environments [35].

## Methods

The study was conducted using a mixed-methods approach combining qualitative media analysis and quantitative analysis of financial data. The aim of this approach was, first, to identify the objectives and justifications connected to EGM reductions in Italy and Finland, and second, to measure the impacts of EGM reductions on total consumption. Financial data reflect the total consumption on EGMs at a population level and therefore are considered to be a suitable proxy for population-level harms.

### Media analysis

The media analysis is based on reporting prior to and during the announced EGM reductions in Italy and Finland. The aim of the media analysis was to analyse and identify (a) the justifications given for the reductions and opposition, and (b) the objectives given in public.

In Italy, the data comprise 17 articles that were collected from the online database of the best-selling newspaper in Italy, *Corriere della Sera* (CS, circulation around 300,000-400,000). In Finland, press items were collected from two main sources. Of the 45 articles analysed, 13 were found through the online database of the largest daily newspaper in Finland, *Helsingin Sanomat* (HS, circulation around 300,000). An additional 32 articles were found using an online database collecting news via RSS feeds of other publicly available Finnish newspapers and media sources (uutispuro.fi, consisting of articles from *Iltalehti* (IL), *Iltasanomat* (IS), *MTV3 uutiset* (MTV3), *YLE uutiset* (YLE) and *Keskisuomalainen* (KSML)). In both countries the items mainly consisted of news pieces, but also included opinion pieces and interviews with relevant stakeholders. Most articles contained characteristics of several types of reporting.

Data were collected using the Italian and Finnish language versions of the keyword ‘gambling machines’ (Italian: *apparecchi da gioco/da intrattenimento, macchinette, slot machine*; Finnish: *rahapelikone, rahapeliautomaatti, pelikone, peliautomaatti*). Data were collected starting 1 year before the announced reductions (in Finland, September 2018, in Italy, September 2016) until the end of February 2020 as subsequent news focused only on the impacts of COVID-19 on gambling. We excluded articles that did not address EGM policy directly.

The data were analysed thematically by two researchers. The researchers discussed the relevant themes based on an initial reading. Finally, the articles were coded by using four thematic categories: (1) Declared aims of policy suggestions to remove or reduce the number of EGMs; (2) Opposition arguments to reductions/removals; (3) Expected impacts of policy changes; and (4) Decisions that were eventually reached.

### Financial data

In the second part, we consider the financial impacts of EGMs in Italy and Finland in terms of total consumption. We accomplished this by looking at (1) the number of EGMs in Italy and Finland before and after (or during) the reductions; and (2) the amount of gross gambling revenue (GGR, i.e., stakes minus winnings) generated by EGMs before and after (or during) the reductions.

In Italy, the figures were drawn from the ADM Libro Blu between 2016 and 2019. The Libro Blu is the official annual report published by Agenzia delle dogane e dei monopoli (ADM, the public branch of the Ministry of Economy and Finance in charge of regulating the gambling market). The financial figures considered were gross gambling revenue and expenditure. The data only concern AWPs as VLTs were not subject to reductions in Italy. As Italian gambling is operated by private multinational groups under concessions, we also investigated how the reductions impacted the share of revenue between the public and the private sectors.

In Finland, financial figures and EGM numbers were requested from the monopoly holder Veikkaus using official data request protocols. As the EGM reductions were set to commence in January 2020, but the COVID-related closures interfered with analysing the impacts after March, we were only able to compare the number of EGMs and the GGR produced by EGMs between January 2018 and February 2020. COVID-19 related EGM closures and possible transfers towards online gambling did therefore not impact the analysis in the current study. The Finnish data on EGMs concerns EGMs in non-casino locations as the reduction policy only targeted these. The data received from Veikkaus had been indexed against the January 2018 baseline by the monopoly holder.

## Results

The results are divided into two parts. First, we considered the justifications and expected outcomes of EGM reduction policies in Italy and Finland using the press material. In the second part, we considered the impacts of the EGM reductions on total consumption using financial figures.

### Justifications and expected outcomes of EGM reduction policies

#### Italy

The debate preceding the EGM reduction policy in Italy followed from a disagreement between the state and the regions regarding whether to adopt a state-level EGM reduction policy. As reductions had already been initiated by municipalities, the debate reflects a conflict of interests between the central and local administrations in Italy. The main stakeholders present in the Italian discussion were therefore not gamblers or gambling providers, but political actors at different administrative levels as well as journalists functioning as watchdogs of political decision-making.

The first article in the discussion (CS 6.4.2016a) reported Prime Minister Matteo Renzi’s announcement to reduce 30% of EGMs, including all non-casino EGMs. The justifications presented by the government were related to the social and health consequences of gambling expansion, as well as the requests of local governments. To reduce the risk of illegal machines, new AWPs would also function on a remote connection. However, on the same date, another article (CS 6.4.2016b) reported a suspicious change in the number of EGMs to be reduced. According to the Italian Stability Law, the calculated reduction of 30% should have been based on the number of machines registered in July 2015 (378,109) instead of the number registered in December 2015 (418,210). This difference was attributed to the lobbying power of the gambling industry. The article also noted that the wide availability of legal gambling does not necessarily prevent illegal gambling − one of the most used justifications by the state to expand gambling [27] – but it does increase state revenue.

The topic was picked up a few days later (CS 12.05.2016) in an article arguing that industry lobbies are *‘*so powerful that they change the course of the law’. In a reply (CS 12.05.2016), the ADM (the Italian monopoly administration) clarified that the December 2015 date referred to the prohibition of issuing new permits for slot machines that are not substitutes for those in use. This claim was followed by a rebuttal by two journalists, arguing that this misalignment enabled the industry to pull over 40,000 old EGMs out of the warehouse, increasing the number of EGMs by 10.6% in 4 months. According to the article, this was another example of corruption in gambling policies. The same was argued in later reporting, pointing out that the stability law has not been followed by any implementing ministerial decree and the actual number of EGMs had increased (CS 16.11.2016; CS 08.04.2017).

In May 2017, discussion on EGMs was re-sparked following a new agreement resulting from the Permanent Conference to reduce the number of gambling venues and EGMs as well as to impose minimum distances between EGMs and sensitive places (such as schools or hospitals) (CS 05.05.2017a; b). The president of the national association of municipalities thought it was *‘a good compromise’* (CS 05.05.2017a). However, in a more critical commentary by a journalist, the agreement is deemed *‘unpresentable’* and unsatisfactory, as the suggested regulation was less strict than the local regulations already in place. The article also points out that ‘anti-gambling movements’ were not satisfied with the agreement. Again, this unsatisfactory result was attributed to industry lobbying. The issue of minimum distances raised further discussion throughout the period researched, mainly from the perspective of local representatives demanding tighter restrictions (CS 09.11.2016; CS 07.07.2017).

Overall, items from 2016 are more openly in favour of heavy EGM reductions and critical of the state and industry lobbies. The need to reduce the number of EGMs was justified in terms of reducing poverty and social inequalities (CS 12.05.2016; CS 29.3.2017) but also the risk of corruption was noted (CS 16.11.2016; CS 08.04.2017).

In 2017, the reporting changed. Individual responsibility for gambling harms was highlighted in an interview about gambling treatment services (CS 28.05.2017), a researcher of the Istituto Superiore di Sanità (Italian National Institute of Health) underlining that ‘*the problem is not gambling itself but the relationship with it’*. The previously critical media appears to have accepted the compromise solution concerning EGM reductions. In the last openly critical item of the analysed period (CS 15.11.2017), a journalist noted that no government, either right or left, or even the media was willing to give up gambling proceeds. This may be part of the reason *Corriere della Sera* did not discuss EGMs further in 2018-2019. Another reason might be the policy decision regarding the extent of national-level reductions, although this is less likely given the important space given to the issue before. Only one press item was retrieved per year during 2018-2019 and neither focused directly on EGMs.

The negative financial outcomes of the reductions appear to have been taken for granted and were not explicitly discussed in the Italian newspaper data. At the same time, other measures, such as tax reform on EGMs winnings to increase state gambling revenue was later introduced (CS 25.10.2018).

#### Finland

Like the Italian case, the developments that led to EGM reductions in Finland followed from a longer discussion on EGM harms, but also from the justification of the Finnish gambling policy [36]. One of the legal justifications for the Finnish gambling monopoly is to prevent and reduce gambling-related harm (Law 2112.2016/1286, section one). However, the aggressive advertising campaigns of the monopoly holder and the placement of EGMs increased critical public discussion during 2018 and 2019 (IL 14.8.2019). In March 2019 (MTV3 20.3.2019), a citizen initiative was launched by experts by experience of gambling harms with the aim to remove EGMs from public spaces. In Finland, citizens can initiate a process for legal change by petition. In August 2019, the Minister of the Interior Maria Ohisalo also suggested looking into removing EGMs from public spaces (HS 12.8., 2019; MTV3, 12.8.2019).

As in Italy, the Finnish media was also initially critical of EGMs. However, unlike the Italian case, the debate in the Finnish press appeared to consider a wider network of stakeholders in EGM policy while the role of journalists remained marginal. The main proponents of removals were citizen activists (MTV3, 20.3.2019), researchers (HS 14.8.2019; HS 16.8.2019), and a few politicians (HS 25.11.2018). Their main argument was to reduce gambling-related harms particularly amongst lower income groups (MTV3, 12.8.2019; HS 16.8.2019). A citizen opinion poll from 31 August 2019, reported widely across the media (IL; KSML; YLE; MTV3; HS 31.8.2019) showed that 60% of Finns were willing to move EGMs from non-casino locations to separate gambling arcades. Some members of parliament (MPs) also supported removing EGMs from public spaces. Based on an opinion poll amongst MPs, those connected to the gambling monopoly Veikkaus were more likely to be against EGM removals than MPs without any relationship with Veikkaus (IS, 17.8.2019). One-quarter of Finnish MPs disclosed a connection with Veikkaus, either as members of the Veikkaus board or via their positions in associations benefitting from revenue generated by Veikkaus (IS, 17.8.2019).

The most vocal stakeholders against the removal of EGMs were representatives of the redistribution network (IL 15.8.2019; MTV3, 16.8.2019; HS 26.8.2019). In Finland, supermarkets, kiosks, and restaurants receive commissions for providing gambling products, including EGMs (YLE 15.2.2020; IL 15.2.2020). The main argument in support of EGMs was the revenue from machines particularly for smaller shops and restaurants and the ensuing effects on employment (IL 15.8.2019; HS 26.8.2019).

Following from this discussion, Veikkaus announced in September 2019 that it would reduce the number of EGMs during 2020 along with other changes to improve its approach to ‘responsible gambling’ (HS 5.9.2019 KSML 5.9.2019). In October, Veikkaus specified that it would remove 3500 EGMs during 2020 and up to 8000 machines in total (IS 31.10.2019; KSML, 31.10.2019). The reductions would be conducted mainly by removing some of the machines from locations with a high number of EGMs (YLE 3.12.2019). The suggestion by Veikkaus was much inferior to the initial suggestions. Furthermore, and in contrast to the Italian case, the reductions did not target the number of resale points. This was probably due to the opposition of resellers. One representative of resellers stated that *‘we now have five machines. You take away the two that make the least profit and replace them with one that makes more. The impacts will be minor’* (IS, 24.2.2020). Some criticism of the limited extent of the proposed reductions emerged from researchers (KSML, 31.10.2019), but reporting on this was limited. The suggestion of the monopoly holder appeared to be accepted in the media without further need for public discussion.

The remaining articles focused on the expected financial outcomes of the reductions. Veikkaus estimated that the planned reductions of EGMs, alongside other changes (reductions in marketing, mandatory identification in 2021) would reduce its annual gross gambling revenue (GGR) by approximately 150-200 million Euros as of 2021 (YLE, 31.10.2019; KSML, 31.10.2019). Reduced total consumption would also impact the revenue of beneficiaries and the redistribution network. Previous research has shown that 60% of Veikkaus GGR is directed to beneficiaries and almost 10% to the resale network [37]. Reductions are estimated to impact almost 2200 resellers (of 6300) (YLE 3.12.2019), some of whom also voiced concern over reduced revenue in media reporting (MTV3, 7.11.2019). Interestingly, impacts on the levels of gambling-related problems or harm were no longer discussed: all discussion on expected outcomes only focused on revenue and beneficiaries.

### Impacts on total consumption

As discussed above, in both Italy and Finland, EGM reductions were justified in terms of public health concerns, but the amplitude of policies varied. In Italy, the aim was to reduce machines by 35%. In Finland, the initial plan was to reduce 18% of non-casino machines, but the aim later increased to 43%. The Finnish reductions did not reduce the resale points, as opposed to a 31% reduction of gambling locations in Italy.

In the following we present available data on the impacts of the reduction policies on total consumption.

#### Italy

**Fig. 1:**
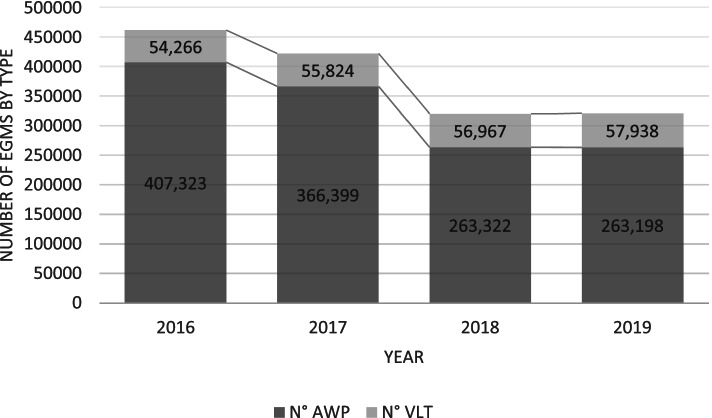
Total numbers of AWPs and VLTs in Italy 2016-2019

**Fig. 2:**
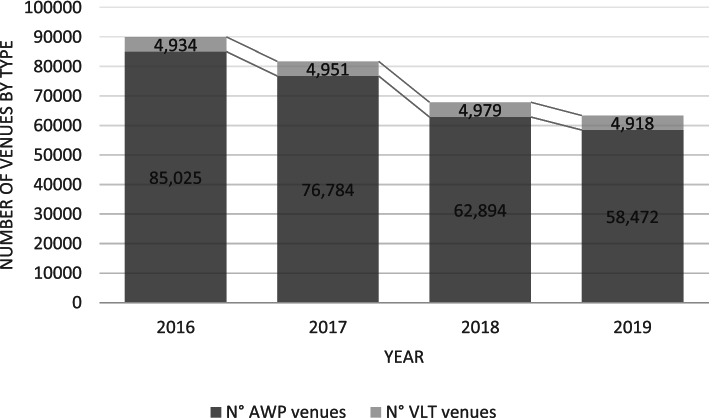
Total numbers of AWP and VLT venues in Italy 2016-2019

The Italian data on the total consumption of EGMs focus on the reductions targeting non-casino AWP machines during 2016-2019. Figure 1 describes the total reduction in machine numbers during the period while Fig. 2 describes the reductions in venue numbers.

As shown in the figures, most reductions took place between 2017 and 2018. Overall, the number of AWPs had decreased by 35% and the number of venues had decreased by 31%, as planned. However, reductions targeting AWPs also translated into a 7% increase in the numbers of VLTs (a structurally more addictive type of EGM confined to gambling arcades). This shift suggests that while the overall numbers of EGMs declined in Italy, some machines were substituted with more addictive modern multigame terminals.

**Fig. 3:**
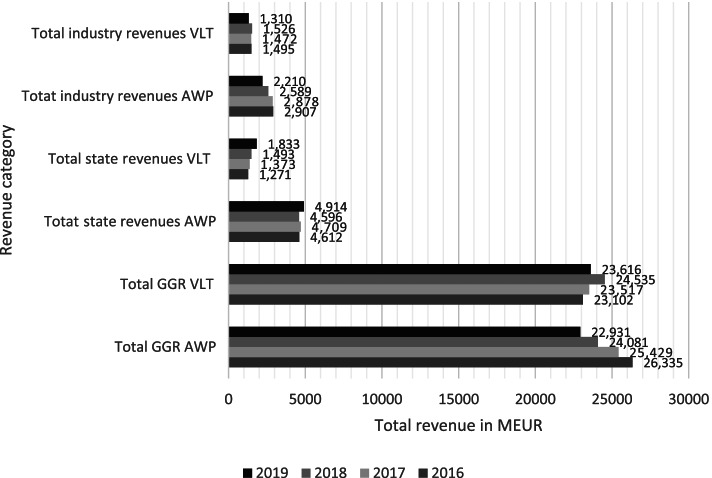
GGR, state, and industry revenue in Italy 2016-2019 in MEUR

Figure 3 shows the impacts of the AWP reductions on total GGR in 2016-2019 as well as the shares of the industry and state revenue as reported in the ADM yearly reports.

The figure shows the main outcomes. First, the GGR, or total consumption, of AWPs decreased in the period 2016-2019 by 13%. The GGR of VLTs increased in the same period by 2%. Overall, the total consumption of EGM gambling therefore declined by only 6%, and not proportionately to the reduction of 35% in machine numbers. A possible explanation for this is that the AWPs targeted for removal were already not profitable and the highest producing machines were left in the market. Furthermore, a substitution effect from AWPs to newer VLTs has compensated some of the loss of GGR, although not fully. Substitution between gambling products is rarely complete. New gambling products substitute some consumption of existing ones, but also increase total market (see also [38]).

Second, state revenue (the surplus raised after operating costs) increased by 7% in the case of AWPs despite reductions, and by 44% in the case of VLTs. This observation also supports the substitution hypothesis. A shift towards VLT gambling increased overall state revenue. The increase in revenue is also explained by a pay-out reduction from 2019 lowering the return percentages of AWPs from 70 to 68% and the return percentages of VLTs from 84 to 83%. In the same period, industry revenues from EGMs declined.

#### Finland

**Fig. 4:**
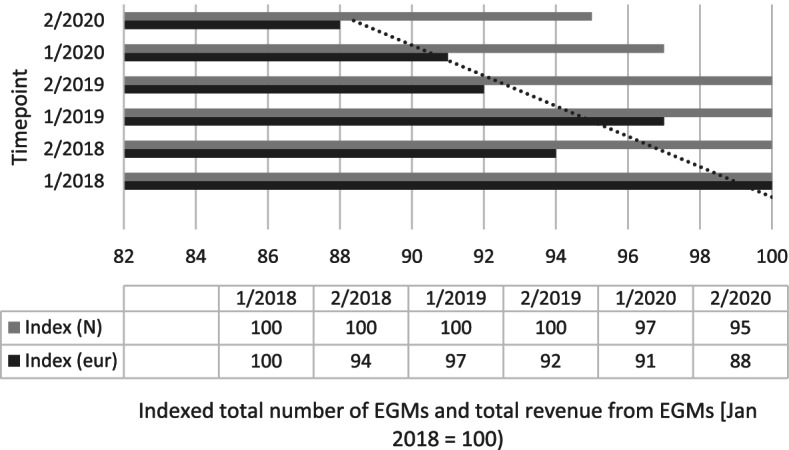
Number and GGR of non-casino EGMs in mainland Finland, January-February 2018-2020

The Finnish data obtained from Veikkaus only covers the initial phase of the reductions as the impacts of EGM removals were confounded by the impacts of COVID-19-related EGM closures after March 2020. The data were indexed by the gambling operator. Figure 4 presents the indexed number of non-casino EGMs and the indexed GGR produced by non-casino EGMs between 2018 and 2020. The number of EGMs and the total GGR from EGMs in January 2018 are indexed at 100 and subsequent data points are calculated to show trends in the change.

The data show that during the first 2 months of 2020, the number of non-casino EGMs had been reduced by 5%. The starting situation indexed at 100 refers to 18,500 EGMs in non-casino locations. The GGR of EGMs had also declined, but the regression suggests that the decline was connected to a longer-term trend of declining EGM gambling rather than the reduction. However, this was expected due to the limited scope of the reductions during the period of analysis. The impacts of the overall reductions could not be analysed with these data due to the onset of COVID-19.

## Discussion

The current study has investigated the justifications, expected outcomes, and impacts on total consumption of EGM reduction policies in Italy and Finland. In both countries, EGM numbers in non-casino locations have been reduced in recent years by approximately one-third. Availability reductions may enhance consumer protection in gambling, but the reductions need to be well-targeted and significant. EGMs are amongst the most harmful forms of gambling and reducing total consumption on EGM gambling and exposure to EGMs in non-casino locations is therefore an important public health intervention. According to the availability theory and total consumption theory, limiting availability should translate into reduced total consumption and therefore reduced harms [4, 14, 15].

According to the data presented in this study, the reductions put in place in Italy and at least in the first months in Finland have not translated proportionally into total consumption. This finding might indicate that the availability theory is not valid, but as evidence from EGM shutdowns during COVID-19, as well as stricter regional reductions in Italy have shown, it is more likely that the reductions in the Finnish and Italian cases have not been significant enough. Criticism of the extent of reductions was also voiced in the media material in both countries. Initially, media discussions in both countries departed from the total removal of all non-casino EGMs but ended in a compromise of more limited reductions. This also limited the extent of public health benefits.

First, in Italy reductions have targeted less profitable machines. One reason for this may reside in revenue interests. In Italy, the state even increased its EGM revenue during the reductions following a tax reform. Targeting less profitable machines may be the case in Finland as well. As discussed above, representatives of the resale network also argued that only the least profitable machines would be removed, similarly to the case of Italy. The Finnish media analysis further suggests that the important revenue interests of resellers and beneficiaries (4000 in total) may have had an impact on how reductions were accomplished.

Second, the reduction in the number of EGMs may not have been significant enough. In the Finnish case, due to data issues we could only analyse the first 2 months of reductions, translating into a decrease of only 5% in machine numbers. However, according to an interview with a Veikkaus representative from 2018, the use rate of EGMs has been only 30% (MTV, 16.8.2019). As the reductions have been targeted at reducing the number of machines in locations that already had a high number of EGMs, the usage rate of the remaining machines may simply increase while no impact will be visible in terms of total consumption. With a usage rate of only 30%, even the anticipated reduction of 43% in the number of EGMs may not have been enough. In Italy, reductions only targeted AWPs and not the more addictive VLTs, the number of which increased during the reduction period.

Third, the number of EGMs alone does not appear to be as relevant as the number of resale points. In Italy, by imposing a minimum distance from sensitive places, reductions also targeted gambling locations. In Finland, the number of locations was not targeted in the policy and exposure to EGMs remained similar in public environments, following the criticism and revenue concerns of the resale network according to the media analysis. No minimum distance from sensitive locations was discussed.

EGMs form a significant part of consumption by problem gamblers in the contexts studied. Public health concerns were also highlighted as justifications in the media discussions on EGM reductions in both countries, albeit alongside concerns about financial results and profits. The media analysis shows that while stricter regulations on the EGM market are initially suggested, the more industry-friendly but less dramatic proposals in the end appeared to be accepted in both countries. This may be due to either lessened public interest but also industry influence. If gambling is to be considered a public health issue, these commercial determinants of health should be addressed [39]. Effective reductions of EGMs inevitably impact company revenue and their monetary contribution to societies. EGMs are purposely designed to be addictive [7]. Limiting access to them is therefore necessary if public health is to be advanced [40].

The current study has been limited to two European countries. However, the cases of Italy and Finland provide relevant results also for other countries and jurisdictions with widespread EGM gambling, such as Australia. As the study has shown, policies to limit EGM harms via availability reductions need to be implemented extensively to achieve reductions in total consumption. As EGM reduction policies have not been widely addressed in previous research, documenting the impacts of policy experiments is crucial to inform decision-makers also in other countries.

The results have also been limited by data availability. More research would be welcome on the impacts of EGM reductions on gambling harms and problem gambling in Italy and Finland, as well as elsewhere. More research is also needed on government reliance on EGM revenue and the coordination of state regulators between the explicitly opposing tasks of revenue generation and the protection of public health (cf. [41]). However, the current study has been able to show the mismatch between justifications, objectives, and implementation of EGM reduction policies in Italy and Finland, and to suggest some ways forward to improve future EGM reduction policies in these or other contexts.

## Conclusion

This study has compared the justifications of EGM reduction policies in Italy and Finland, as well as the effects of reductions on total consumption. The results have shown that despite ambitious initial suggestions to remove or reduce the number of EGMs in both countries, the public debate and industry lobbying resulted in lukewarm solutions. Financial figures indicate that the reductions may not have been significant enough in either country to reduce the burden of EGMs on public health efficiently.

## Data Availability

The data underlying Fig. 4 are available from the Finnish gambling monopoly operator but restrictions apply to the availability of these data. An official data request should be made to Veikkaus to gain permission for its use. Other data are available from public sources and can also be requested from the authors.

## Abbreviations

ADM: Agenzia delle dogane e dei monopoli
AWP: Amusement with prizes
CS: Corriere della sera
EGM: Electronic gambling machine
GGR: Gross gambling revenue
GRET: Gambling restraint erosion theory
HS: Helsingin Sanomat
IL: Iltalehti
IS: Iltasanomat
KSML: Keskisuomalainen
LDW: Losses disguised as wins
MEUR: Million euros
MP: Member of parliament
VLT: Video lottery terminal
